# Could Experimental Inflammation Provide Better Understanding of Migraines?

**DOI:** 10.3390/cells11152444

**Published:** 2022-08-06

**Authors:** Philip Victor Reducha, Lars Edvinsson, Kristian Agmund Haanes

**Affiliations:** 1 Department of Clinical Experimental Research, Glostrup Research Institute, Copenhagen University Hospital, Rigshospitalet Glostrup, 2600 Glostrup, Denmark; 2 Department of Biology, Section of Cell Biology and Physiology, University of Copenhagen, 1017 Copenhagen, Denmark; 3 Division of Experimental Vascular Research, Department of Clinical Sciences, Lund University Hospital, 221 00 Lund, Sweden

**Keywords:** inflammation, migraine, CGRP, CFA, inflammatory soup

## Abstract

Migraines constitute a common neurological and headache disorder affecting around 15% of the world’s population. In addition to other mechanisms, neurogenic neuroinflammation has been proposed to play a part in migraine chronification, which includes peripheral and central sensitization. There is therefore considerable evidence suggesting that inflammation in the intracranial meninges could be a key element in addition to calcitonin gene-related peptide (CGRP), leading to sensitization of trigeminal meningeal nociceptors in migraines. There are several studies that have utilized this approach, with a strong focus on using inflammatory animal models. Data from these studies show that the inflammatory process involves sensitization of trigeminovascular afferent nerve terminals. Further, by applying a wide range of different pharmacological interventions, insight has been gained on the pathways involved. Importantly, we discuss how animal models should be used with care and that it is important to evaluate outcomes in the light of migraine pathology.

## 1. Pathophysiology of Migraine

Migraine is a common neurological and headache disorder affecting around 15% of the world’s population (≈1 billion people, reported migraine in the previous year) and are three times more prevalent in women (around 18%) than in men (around 6%) [1,2]. It is the second cause of disability worldwide (behind only lower back pain) and the leading cause of neurological disability. Migraines share some similarities with other primary headache disorders in multiple aspects which can lead to misdiagnosis, but migraine also have several unique features. Various probable migraine triggers have been identified including genetic factors such as channelopathies that are familial or sporadic: maternal inheritance of mitochondrial encephalopathy, lactic acidosis, and stroke-like episodes; angiopathy inheritance, to name a few [3].

For a long time, the vascular theory of migraine was dominant. The theory was coined by Harold Wolff in the 1940s. He hypothesized that vasodilation of cranial vessels was the cause of migraines [4]. However, the vascular theory of migraine has since been challenged and by many replaced by neuronal theories which include the central and/or the peripheral nervous systems in migraine pathophysiology. Nevertheless, the vasculature has remained an important factor in migraine pathophysiology as the neurovascular theory of migraine is currently dominating. The neurovascular theory suggests involvement of the trigeminovascular system (TGVS) in the ictal phase of a migraine attack [5]. Neurogenic inflammation (NI) has also been proposed to play a part in migraine chronification. NI would consist of vasodilation, mast cell degranulation, plasma protein extravasation, platelet aggregation and the involvement of vasoactive peptides and inflammatory mediators such as calcitonin gene-related peptide (CGRP), substance P, and neurokinin A, as well as of prostaglandins [6]. However, besides CGRP, the involvement in migraine pathophysiology of the other neuropeptides, as well as mast cell degranulation and plasma extravasation is unclear and needs further research [7]. Peripheral and central sensitization have been proposed as likely causes of pain and involved in the process of chronification of migraine [8].

Sensitization is the process where the response to a stimulus is facilitated and the response itself increased in magnitude. The activation of endogenous inflammatory mediators (which are yet to be fully recognized) is believed to sensitize peripheral trigeminovascular neurons (or first-order neurons) through constant innervation of the dural meninges. Peripheral sensitization likely causes the throbbing nature of the headache, and CGRP has been observed to be involved in initiating and sustaining peripheral sensitization. The constant stimulation of the peripheral TGVS causes sensitization of second-order neurons (trigeminocervical complex, trigeminal nucleus caudalis (TNC)), third-order neurons (trigeminothalamic), as well as neurons in the brainstem and thalamus, causing central sensitization. Central sensitization may cause muscle tenderness, aversion to touch, as well as cephalic and extracephalic allodynia and could possibly be the reason that migraines turn chronic [9,10]. The neurovascular theory builds on the view that vasodilatation of cranial arteries excites the sensory perivascular nerves which cause either immediate or delayed headache pain [11]. The view suggests that vasodilatation activates cyclic nucleotides and potassium channels, resulting in a local rise in potassium ions which causes activation of the sensory nerve terminals in the dura mater [12].

The initiation of migraines is also unclear but likely occurs centrally, as fMRI scans have shown increased activation of the hypothalamus during the prodrome phase [13]. Cortical spreading depression (CSD) has been proposed to play a significant role in the initiation of a migraine attack. However, only 25% of migraineurs experience aura symptoms [14]. In migraines without aura (MoA), if a minor reduction in brain blood flow occurs it is not observed with current methods. Therefore, arguments have been made that CSD only causes aura in patients that have a specific genetic predisposition, which would explain the lack of experience of aura symptoms in MoA patients. Therefore, the role of CSD as a general migraine trigger has been questioned [15]. Regarding CSD and inflammation, there is no evidence of the depression itself being caused by inflammation. However, experimental CSD can trigger neurogenic meningeal inflammation and subsequently activate the trigeminovascular system [16,17]. However, this has not been confirmed in humans.

Although the origin and cause of migraine is not fully known, CGRP is an established key component in migraine attacks. For example, the plasma levels of CGRP are greatly increased during migraine attacks [18], and inhibition of CGRP release decreases both plasma levels of CGRP and the severity of migraine symptoms [18,19]. The role of CGRP in migraines is associated with CGRP being a strong vasodilator and involved in pain transmission from various parts of the head to the central nervous system. Therapies targeting CGRP release or its canonical receptor in the TGVS have been proven to ease symptoms and alleviate pain [5,20,21]. Binding of CGRP to its receptor (between RAMP1 and CLR) leads to conformational changes of the CGRP receptor, and activation of the Gα_s_ subunit. The activated Gα_s_ subunit binds to adenylate cyclase (transmembrane protein), which converts adenosine triphosphate (ATP) into the second messenger known as cyclic adenosine 3’,5’-cyclic monophosphate (cAMP). Intracellular cAMP levels are increased and causes an intracellular downstream signalling cascade. The increase in intracellular cAMP activates cAMP-dependent protein kinase A (PKA) signalling, which results in the activation of cAMP response element-binding protein (CREB), which finally affects gene transcription [22]. In the context of migraines, the increase in intracellular cAMP levels has been suggested to cause opening of hyperpolarization-activated cyclic nucleotide gated channels (HCN) or ATP-sensitive potassium (K_ATP_) channels that could lead to hyperexcitability of the membrane and possibly be the reason for migraine pain [23].

CGRP has been observed in regions of the central nervous system (cortex, thalamus, hypothalamus, TNC etc.) [24,25]. Additionally, CGRP release has been linked to peripheral areas relevant to pain sensation during the ictal phase of migraine, such as the dura mater and trigeminal ganglion (TG) [18,26]. CGRP and the CGRP receptor are localized in proximity and in parallel to each other, as CGRP is found in great numbers in unmyelinated C-fibers, while CGRP receptors are localized in the Nodes of Ranvier of myelinated Aδ-fibers, both in the TG and dura mater [27]. The TGVS, where CGRP is found in high concentrations, consist of the trigeminal nerve (or fifth cranial nerve, CN V) which has three distinctive branches: the ophthalmic (V1), the maxillary (V2), and the mandibular (V3) branch. As reviewed by Walker and colleagues [28], V1 innervates and supplies strictly sensory information to intracranial dura matter and brain vessels as well as extracranially to the upper facial areas forehead and the scalp, and to orbit and the eyes, upper nasal cavities and skin above the eyes and upper parts of the nose. V2 innervates and conveys strictly sensory information to mid-facial regions, including inferior nasal cavities and sinuses, as well as the upper jaw. V3 innervates sensory information and has motor functions by innervating the muscles of lower facial areas, and the mandibles. In the context of migraines, it is believed that nociceptive information caused by the excitation of C-fibers and in turn of Aδ-fibers, passes along the trigeminal nerves (V1, and to lesser extend V2-V3) in a series of action potentials and merges in the TG. From the TG, the sensory information is sent further to second-order neurons in the trigeminocervical complex (TCC which contains: TNC, dorsal horn and upper cervical spinal horn C1-C2). The nociceptive information is passed on deeper into the central areas and processed by the nuclei of the brain stem, the thalamus and the hypothalamus. Visual, auditory, olfactory, somatosensory, and motor cortices receive the nociceptive signals and are believed to cause the characteristic migraine symptoms such as phonophobia, photophobia, headache pain or cognitive dysfunction [9].

CGRP is believed to play a big role during this communication of pain information [5,29]. It is postulated that during the ictal phase of migraines, neurons in the dura mater and other peripheral neurons receive increased stimulation causing the release of CGRP from C-fibers. CGRP binds to the CGRP receptor located in the Aδ-fibers, which further stimulates neurons, causing extended depolarization. Depolarization and CGRP release are continued along the trigeminovascular pathway, creating nociceptive signalling. The initial release of CGRP in the periphery is likely caused by the excitation of neural membranes from action potentials or through antidromic signalling, causing an opening of ion channels such as voltage gated sodium (Na^+^) ion channels. The opening of voltage-gated ion channels leads to an influx of calcium ions (Ca^2+^) into the cytosol provoking exocytosis of CGRP from vesicles and into the synaptic cleft; that CGRP can later be detected in blood samples from migraine patients [18].

## 2. Inflammation in Migraine

**Figure 1:**
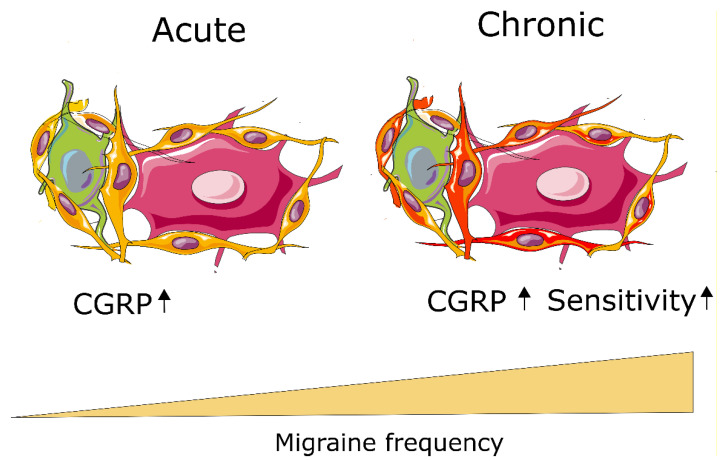
**Chronification of migraine.** Small CGRP-containing neurons (green), and larger CGRP receptor-containing neurons (pink) are surrounded by satellite glial cells. We postulate that activation of the satellite glial cells (indicated by a change in color from orange to red) sensitizes the glia/neuron subunit, thereby decreasing the migraine threshold and leading to increased migraine frequency.

During the years there have been much work, mainly in rodents, suggesting a significant role of NI in migraines. Overall, the current view is that in single migraine attacks there is limited evidence of inflammation. However, many migraine cases evolve over time to be more severe and even enter a chronic phase. It is therefore possible that inflammation might play a role in migraine chronification, which is the current working hypothesis (Figure 1). To understand such a notion would assist in providing better therapy.

In the 1980s, the theory “sterile neurogenic inflammation” of the dural meninges was presented [30]. The involvement of inflammation in migraines was further supported by preclinical studies that showed significant inhibition of plasma protein extravasation with classic anti-migraine drugs, ergots and sumatriptan [31,32,33,34]. Considerable evidence suggested that NI in the intracranial meninges could be a key element resulting in the sensitization of trigeminal meningeal nociceptors in migraine [35,36,37,38].

Major cytokines have been implicated in the TGVS pathway resulting in NI, including tumor necrosis factor (TNF)-α, IL-1β and IL-6 [39,40]. There are studies that observed inflammatory molecules, in particular TNF-α, in plasma, serum or urine samples. These data suggested presence of inflammation in patients when comparing the levels during attacks and in attack-free intervals [41,42]. In animals and particularly in the knock-in animal model of familial hemiplegic migraine type-1, it has been shown that mRNA expression of TNF-α was increased in the TG [43].

Elevated TNF-α serum levels in humans were found even outside of attacks, supporting a role of TNF-α in migraine [44] the. However, data is variable as some have shown that TNF-α is only elevated immediately after the attack and is lower in-between attacks [42]. For other cytokines, such as IL-1β and IL-6, these were reported to be increased during a migraine attack [45]. Further, an increase in IL-6 and TNF-α has been detected in the jugular venous blood during a migraine attack [46]. Although changes in TNF-α and other cytokines can be confirmed, it is not clear as to their significance in migraine pathology. In migraine patients, there is often an increase in inflammatory markers over the course of the disease.

As CGRP might be released during the 72 h of the migraine attack [18], it could potentially evoke continuous activation of C- and Aδ-fibers. This in turn would lead to an increase in expression of inflammatory cytokines, not only in the dura mater, but possibly also in neuronal cell bodies, which are localized in the TG and the TNC. Here the term “neurogenic neuroinflammation”, which is defined by inflammatory reactions in the nervous system in response to neuronal activity, can be applied [47]. Combined with central effects which play an important part in causing pericranial allodynia, hyperpathia and muscular pericranial hyperactivity, part of the pathology could also be linked to the peripheral effects of CGRP (or inflammatory sensitization). For example, migraine-proving agents generate periorbital allodynia, for example in animals [48,49]. This is likely due to the release of inflammatory mediators (bradykinin, prostaglandins, etc.) from nerve endings or cells of the immune system [50,51,52]. Worth noting is that peptidergic fibers were not observed along or in between the striated muscle bundles of the temporal or the occipital regions, only in the skin or accompanying the blood vessels [53]. Furthermore, a part of this sensitization could also occur at the nodes of Ranvier [27]. How the nodes of Ranvier could relate to inflammation remains to be explored. There are several studies that have utilized the case that migraines are linked to meningeal/dural activation of nociceptive fibers. Data from these models supports that sensitization of trigeminovascular afferent nerve terminals. This has been studied using several different methods and particularly electrophysiology has given insight into changes in meningeal excitability [54], and has provided insight in modulation of ion channel excitability [54,55] and responses to migraine therapeutics [55]. Data using an inflammatory model has given evidence that triptans can induce hyperpolarizing shifts in meningeal nociceptive fibers [55]. Further, some sex specific difference can also be observed using this model, even in electrophysiological properties [56], adding value of this model in relation to sex differences in migraine pathology in humans.

## 3. The Inflammatory Soup (IS) Model and Its Variants

The most common inflammatory migraine model is to surgically insert a cannula that reaches the dura of anesthetized animals and inject inflammatory mediators through the cannula. This model was first applied by Oshinsky and colleagues [57] and has been applied in various migraine research papers since. Certain variations of the model have been used, notably in the type of animals used, changes in surgical procedures or variations in injection volume, time points and frequency. Besides using cannulas, some groups use supradural catheters [58].

The application of chemical stimulants through this model and its variants has led to various physiological and behavioural changes in animals. These findings allow us to evaluate the relevance of this model as a tool for migraine research and act as reference points if one desires to use this model. Here we will present some of the main findings. First, we look at the effect of IS. IS applied to the dura causes periorbital and/or plantar allodynia [57,58], however most often the allodynia is observed after chronic administration. Additionally, other behavioural impairments have been observed. There are reports of increased face rubbing [59] and grooming [60] which are indicative of head pain and/or irritancy. Furthermore, increased rest and freezing behaviour, and decreased exploratory behaviour have been reported [61], which are behaviours also experienced by migraine patients. On the contrary, Liu and colleagues [62] observed increased distance travelled by IS rats in the Open Field Test (OFT), which was argued to be due to higher anxiety levels.

Some other notable findings are the changes at the cellular level, and we have summarized all the below findings in supplementary tables, notably the effects in TG (supplementary Table S1), brain/spinal cord (supplementary Table S2) and TNC (supplementary Table S3). The levels of c-Fos, a marker of neuronal activation and nociception, have been observed at higher expression levels in the TG and TNC [63,64,65]. Increases of other nociceptive and neuronal activation markers in the TGVS have been observed, such as increases in cAMP [64], PKA [64], ERK_1/2_ [64], CREB [64], NGF [66], PKC [66], tyrosine receptor kinase B (TrkB) [67] and EAAT3 [67]. Increases of inflammatory markers have also been observed in the TGVS, notably an increase in IL-1β [65,68], IL-18 [69], TNF-α [65,70], p38 [67], BDNF [67], Iκβ [71], TRIF [71], TLR4 [71], Myd88 [71] and NF-κβ [71], FKN [72], CX3CR1 [72] and PGE_2_ [73]. Also, an increased presence of microglia [59,67,69,70,71,74,75] and astrocytes [69,74,75] has been reported. As mentioned previously, releases of neuropeptides such as CGRP, substance P and PACAP-38 are notable components of NI and these peptides are also linked to migraine pathophysiology, especially CGRP. Chronic application of IS on the dura causes upregulation of CGRP genes [62,63,66,67,73,75,76,77,78,79,80,81,82,83] and Substance P [76,80,81,82] in the TGVS. PACAP-38 is increased in the plasma of migraineurs during attacks but reduced in chronic migraineurs [84,85]. Long-term IS infusion decreased PACAP-38 which the authors argue is due to PACAP being depleted after chronic administration [86].

Other notable pathological alterations caused by IS application to the dura include a decrease of 5-HT in the brain [65], an increase in the 5-HT_7_ receptor expression [64], a decrease in α7nACh receptor expression in the hippocampus [75], an increase in nitric oxide (NO) [79], increases in the warm receptor TRPV1 [65] and cold receptor TRPM8 [87] expression, decreases in SIRT1 and PGC-1α [80], mitochondrial dysfunction [80,88], an altered functional connectivity between various brain regions [68,89,90,91,92], an increase in EphB2/EphrinB2 receptors [81], increased synaptic plasticity in TNC [76,79,81] but decreased plasticity in the hippocampus [93], an increase in glutamate in the TNC [94], an increase in mGluR5 [95], a decrease in GABA [94], decreases in GABABR1 and GABABR2 [94], increases in mTOR and autophagy [95], an increase in ASIC3 receptor expression [66,82], the increase of nNOS in the brain of rhesus monkeys [83], the impairment of descending inhibitory pathways [96], an increase in nerve growth factor (NGF) [66], increases of the P2Y_14_ receptor [59] and the P2X_4_ receptor expression [67], an increase in BBB permeability [74,97], the increase of VEGF [97], the sensitization of trigeminovascular neurons [96,98,99,100,101], an increase in white matter in different brain regions [102] and changes in the gut microbiota [65]. The application of other chemical stimulants that are inflammation-relevant have also been investigated. A chronic dural application of IL-6, a pro-inflammatory cytokine increased in the blood and serum of migraineurs [103], has been reported to cause periorbital and plantar allodynia [104,105,106,107], upregulate ERK1 [107] in the TG and sensitize dural afferents [107].

Treatments that can reduce the pathological changes of the chemical stimulants may give indications of what could be potential migraine treatments and could be used as positive controls in future studies that will use this model. Treatments that managed to reduce periorbital and/or plantar allodynia or other pathologies induced by IS application to the dura include sumatriptan [65,97,108], ketoprofen (NSAID) [73], nimesulide (NSAID) [73], etoricoxib (NSAID) [73], baclofen (GABA_B_ receptor agonist) [95], propranolol (β-blocker) [101], amitriptyline (tricyclic antidepressant) [93], minocycline (tetracycline-derived antibiotic) [70], rapamycin (mTOR inhibitor) [95], anti-IL18 [69], anti-NGF [66], flunarizine (calcium channel blocker) [62], H89 (PKA inhibitor) [94], chelerythrin (PKC blocker) [66], APETx2 (ASIC3 inhibitor) [82], MPEP (mGluR5 antagonist) [95], PNU-282987 (α7nACh receptor agonist) [75], TNP-ATP (P2X inhibitor) [67], ibudilast (PDE inhibitor) [70], electroacupuncture [64,100,109], EphB1-Fc (EphB receptor inhibitor; only 0.5 µg) [81], TAK-242 (TLR4 inhibitor) [71], naltrexone (TLR4 antagonist) [70], PP2 (NR2B-pTyr inhibitor) [76], genistein (protein tyrosine kinase inhibitor) [76], ANA-12 (TrkB antagonist) [67], SIRT1720 (SIRT1 activator) [80], xiongmatang extract [62], and wuzhuyu decoction [65]. Meanwhile, treatments using APETx2 [104], ANA-12 [104], anisomycin (protein synthesis inhibitor) [110], 4EGI-1 (translation initiation inhibitor) [110] or U0126 (MEK inhibitor) [107] alleviated IL-6 induced periorbital/plantar allodynia.

## 4. Dural Activation by Complete Freund’s Adjuvant (CFA)

We have, in addition to experiments with IS, used additional preclinical models involving inflammatory stimulation of the peripheral parts of the trigeminal system to simulate the chronic nature of migraines without receptor activation. Firstly, we studied inflammatory pathways in cultured rat TG cells [111,112]. Secondly, we administered CFA (Complete Freund’s Adjuvant) into the temporomandibular joint (TMJ), which elicited activation of TG [113]. Thirdly, we developed a new animal model for trigeminal activation using chemical stimulation of the dura mater with CFA [114] to test whether application of CFA on a small part of the surface of the dura mater can cause long-term activation of the TG, and thus provide a model of migraine chronification [115]. Lastly, we evaluated whether activation of TNC and central sensitization occurs following CFA-induced activation of the dura mater [116].

Using cultures of isolated trigeminal neurons as a model for studies of neurons and glial cells, we found enhanced expression of CGRP in both neurons and satellite glia cells (SGCs) following inflammation. One of the most notable changes was in the mitogen-activated protein (MAP) kinase phosphorylation. The findings indicate that activation of a MAP kinase–dependent inflammatory signal pathway is involved in over-expression of CGRP in nociceptive neurons and could participate in generating pain hypersensitivity [117]. Looking further into in vivo inflammation, we showed that administration of CFA into the TMJ elicits activation of TG by increased expression of pERK1/2 (phosphorylated extracellular signal-regulated protein kinase), pp38 (phosphorylated p38 MAPK/ERK signaling pathway), CaMKII (Calmodulin-dependent protein kinase II), NF-κB and DREAM (Downstream regulatory element antagonist modulator) after 2 and 10 days. By applying CFA to induce local inflammation in the TMJ, this caused an upstream inflammation response in the TG where the TMJ sensory fibers have their cell bodies [113].

The inflammatory response does not only involve neurons, but importantly also SGCs, which together represent one anatomical and functional unit [113]. The idea of stimulating the nerves with inflammatory mediators was first shown by Takeda and colleagues, who injected CFA in the TMJ, and detected trigeminal activation. Importantly IL-1 receptor increased in the TG neurons, and this was combined with a potentiated excitability of Aδ-/C- fibers [118]. Furthermore, they later showed that the increased Aδ-fiber activity caused by inflammation could be blocked by an IL-1 receptor type 1 antagonist [119]. Others have shown that application of inflammatory substances onto the dura mater or chemical stimulation of the dural receptive fields causes hypersensitivity to mechanical and thermal stimulation together with direct activation of the TG [120].

The main application on the inflammatory investigation in rodents was the idea of applying the local inflammation of dura mater nociceptive fibers and the following activation in the TG. In the first papers we showed that the application of CFA [120,121] onto the dural surface indeed activated the TG, with the most remarkable changes seen in the expression of pERK1/2, IL-1β and CGRP positive nerve fibers in the TG [115,122]. This illustrates that the application of CFA onto the dura mater could be used as an animal model for long-term activation of the TGVS [115]. We further used this model to show that the application of CFA also induced activation (increased expression of c-Fos) of the central part of the TGVS: the TNC and C_1_-C_2_ regions of the spinal cord [116]. Interestingly, this inflammation could be blocked by the administration of a kynurenic acid analogue (SZR72), which is a precursor of an excitotoxin antagonist and anti-inflammatory substance [116,122]. Linking CFA to neurogenic neuroinflammation, masseteric injection of CFA caused spontaneous orofacial pain behaviors, neuronal activation in the TNC, and the release of interleukin-6 (IL-6). In this short-term study, pretreatment with a CGRP antagonist reduced pain behaviors. Nevertheless, IL-6 was unaffected by MK-8825 [123] but the study would probably have benefitted from a longer time frame than the 24 h used.

## 5. IS vs. CFA

Questions should also be asked about what type of inflammation model would best fit a migraine model. There are some notable differences between the IS and CFA. Usually, the IS consists of inflammatory mediators endogenous to what the body would release during an immune response like histamine, bradykinin, prostaglandins E2 and serotonin, and they are usually mixed into acidic PBS. Meanwhile, CFA is a water/oil emulsion consisting of dried and inactivated mycobacteria (most often *Mycobacterium tuberculosis*). IS induces a sterile inflammatory environment while CFA can go beyond and involve the adaptive immunity as well [124]. CFA is therefore generating local inflammatory markers, without direct receptor-induced activation which is opposite to IS.

One could argue that in the case of most migraines, it is more likely that a sterile inflammation is occurring caused by tissue damage and repair mechanisms initiated from prolonged neuronal activity and surplus of energy used, giving the upper hand to IS as being more relevant in migraines. However, CFA should not be dismissed, as CFA has been reliably used for multiple inflammation and pain models, and as mentioned earlier CFA creates similar cellular and sensitizing effects as IS when applied to the dura [115,116,125]. Further, one could question if some components of the IS could lead to indirect release of other signaling molecules such as ATP [126,127]. In addition to reactions to histamine and bradykinin, this has also been reported for other compounds known to induce itch [128,129] or pain [130]. Secondary effects from ATP could then affect mast cells [131], sensory neurons [132,133] or for example stimulate the release of CGRP [134], further complicating the interpretation of the data.

One could also consider whether the recipe in the IS should be revised, adding some inflammatory mediators that have been observed in the serum of migraine patients like IL-6, CGRP and TNF-α. Arguments could be made whether 5-HT should be removed from the IS mixture as 5-HT have potential anti-migraine effects, as triptans and ditans are agonists of 5-HT_1B/D/F_. receptors. However, as mentioned earlier, applying IS reduces 5-HT in the TGVS in one paper [65] and it is still uncertain whether 5-HT levels fluctuates in migraineurs [135]. Another aspect to take under consideration when using an inflammation model, is the possibility of surgical procedures creating inflammation on their own, and so using SHAM animals should be highly considered. In addition, anaesthesia, antibiotics and painkillers used for recovery could potentially have a modulatory effect on the induced inflammation.

## 6. Evaluating Outcomes in Animal Inflammation Models

**Figure 2:**
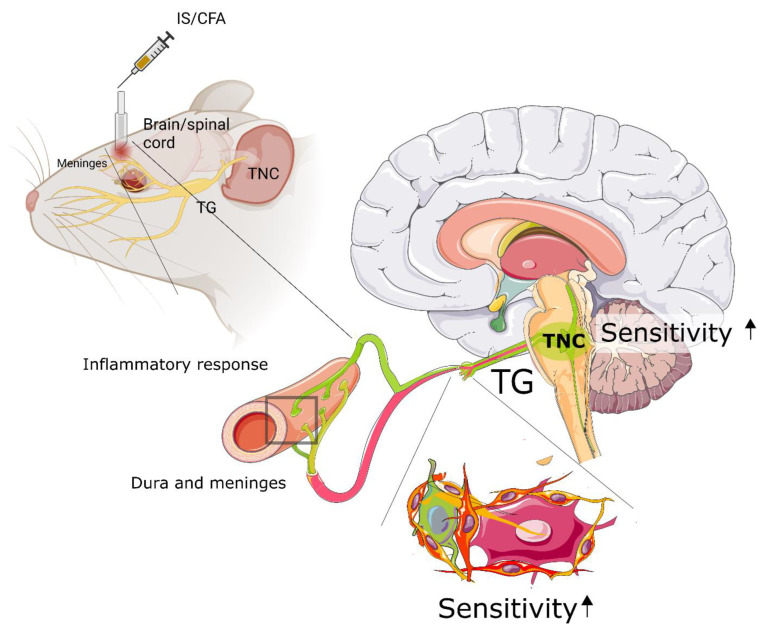
**Sensitization in animal models.** The chronification of migraines can to some extent be mimicked by the application of inflammatory substances such as Inflammatory Soup (IS) or Complete Freund’s Adjuvant (CFA) to the dura and meninges of rodents. Experimental data suggest that this activates satellite glial cells (indicated by a change in colour from orange to red) sensitizes (black arrow) the trigeminal ganglion (TG), and the trigeminal nucleus caudalis (TNC).

The final goal of the animal research into migraines and the inflammation aspects thereof is to gain mechanistic insight into the pathology, we therefore address some important considerations when evaluating outcomes in inflammation studies. It is important when looking for translational aspects and identifying novel therapeutic targets that the model and outcomes represent true relatable outcomes. The main concept is that inflammatory stimulation of the dura will affect the nociceptive input from the meninges, with a headache most likely occurring in the animals due to the activated nociceptors from the meninges (Figure 2).

The inflammatory approach usually includes a craniotomy, which is an invasive procedure, might have relevance in interpreting the data. As an example, the paper by Laborc and colleagues highlights that the mere attachment of a rat in a stereotaxic frame can activate the trigeminal system [136]. This highlights the additions made by using both fresh controls and also shams in the uncovering of the true role of inflammation, and its separation from the invasive measures [137]. As we have seen above, some of the endpoints are similar as seen in human migraine attacks, but these studies will always be limited by the lack of direct communication with animals. Below, we discuss some limitations linked to outcomes in animal models.

Although photophobia is a known symptom of migraines [138], the general mechanism of migraine-induced photophobia is still unclear. The convergence of optical signals from retinal photoreceptors and nociceptive signals from the TGVS to the CNS is the most likely cause of photophobic experiences in migraineurs [139]. Investigating photophobia is generally a hard task due to the lack of well-established investigation tools and the naturally light-aversive behaviour of laboratory rodents as they are anxious about being seen by predators [140]. Nonetheless, a Light/Dark (LD) box test has previously been used in migraine research [141].

The LD box test does not exclusively capture photophobic behaviour, as it also can capture anxiety-like behaviour and measure locomotor activity [142]. A parameter like “time spend in light zone” is the most indicative of photophobic behaviour. In most studies to date, no difference was observed in similar LD box tests, in rodents where CFA has been administered [143,144]. However, CFA administration in those studies were not used in the context of migraine research and were targeted at inducing inflammation in the plantar region. Studies using other inflammatory mediators, such as NTG, have reported more light-aversive behaviours in NTG treated rodents compared to untreated rodents [145].

Other known behavioural migraine symptoms include anxiety and aversion of physical activity because of experienced pain [146]. The OFT has been reported to capture exploratory and anxiety-like behaviour, as well been able to measure locomotor activity [147]. One of the strongest arguments for including this outcome is the concept that physical activity is negatively influenced by headaches, which could be detected in the lack of exploratory behaviour. This is exemplified by part of the diagnostic criterion for migraines, where the headache is supposedly worsened by physical activity, or that the patient will avoid activity [148].

Although like the LD box test in this regard, the OFT differs by having a larger area for exploration and the lack of a high-intensity light. Anxious or depressed rats are expected to spend less time in the centre zone, while worse locomotor and exploratory performances are more associated with experiencing pain. Care should be taken with sequential testing as the animal would lose the novelty and exploratory features of OFTs, admittedly causing inconsistency in performances and/or defeating the purpose of the test. Either way, different strategies should be approached before initial tests to make sure to produce the most consistent results possible.

Previous migraine studies reported both periorbital and plantar allodynia when rodents were administered with migraine factors to the dura, such as IS [78,149]. The main question is whether the Von Frey threshold is capturing headache and not a cutaneous hypersensitivity, as both are seen in migraine patients. One important issue is that allodynia is not headache, and therefore therapeutic approaches might lead to treatments for allodynia and not for headache.

## 7. Concluding on CGRP and Inflammation—A Perspective

It is no surprise that inflammation is believed to play a potential part in migraine pathophysiology when CGRP is both a key player of migraine pathophysiology and NI. However, it is still unclear whether CGRP is an inducer of inflammation or a by-product of it. Subsequently, the question arises whether inflammation follows the activated areas in migraines (for example is caused by CGRP) or if inflammation causes the activation (and CGRP release). Nevertheless, one would postulate that anatomical separation between the hemispheres would occur, to give rise to the one-sidedness of migraine pain. CGRP even exerts anti-inflammatory properties in some tissue, questioning its role as a pro-inflammatory mediator and its safety as a systemic drug target, as blocking the anti-inflammatory properties could result in inflammation-related consequences elsewhere [150].

Neuroinflammation is typically viewed as a protective response to tissue damage. The main question in migraine is therefore also what type of tissue damage could lead to this initial tissue damage. One possibility could be a neuronal energy deficit. Indeed, this has mainly been put forward by Borkum [151], in light of data on a suggested energy deficit in migraines, particularly by a lower phosphocreatine-to-creatine ratio and an increased concentration of ADP [152,153]. In addition, migraines are associated with mitochondrial disease [154] and with subjects undergoing a fasting state [155]. One potential explanation could be a link to increased membrane potential as the cells struggle to maintain the resting voltage, which could potentially lead to CSD [156] or increased sensitivity in peripheral nerve fibers [27]. Following a potential energy deficit, an increase in inflammatory markers would be expected to protect the brain [157]. This neuroinflammation would control the severity and possible progression, but could also have detrimental effects [158]. The following neuroinflammation has in other systems been showed both to induce and aggravate neurodegeneration, but at the same time aids in the recovery of neurons [159]. This balance is clearly important when addressing inflammation as a potential clinical target.

Nonetheless, using CGRP-targeted drugs on inflammation models could inform us both of the role of CGRP in inflammation and whether inflammation plays an important role (if at all) in migraine pathophysiology. From what we could gather, the effects of anti-CGRP on IS and CFA have yet been investigated. However, triptans appear to mitigate the effects of both IS [65,101,105] and CFA [160,161], but whether it is due to their CGRP targeting or separate anti-inflammatory effects is not clear. Similarly, α-CGRP_(8-37)_, a CGRP receptor antagonist, was able to reduce allodynia from intraplantar CFA [162]. Mixed data exist on the effect of gepants on CFA. The gepant BIBN4096BS (olcegepant) applied topically, intravenously but not spinally [163], was able to alleviate inflammatory pain from subcutaneously plantar administered CFA. However, intrathecal administration of BIBN4096BS did not reduce the increased nNOS that intraplantar CFA caused [164].

Interestingly, when applying CGRP unto the dura mater, it only causes hypersensitivity in female rodents, at least at doses up to 3.8 μg [106], which could be caused by differences in oestrogen signalling [165]. Therefore, if one was to test the efficacy of CGRP-targeting drugs in the inflammation models, one should be wary to also include female rodents if no effects of the drugs is found in the males. An increase in CGRP release at baseline values or stimulated dura mater compared to fresh rats could indicate a sensitized dura mater. Additionally, a significant reduction in CGRP release could indicate a desensitized dura mater from repeated stimulation. To our knowledge, none of the inflammatory models have tested this outcome. An obesity model showcased an increase of basal CGRP in the dura in rats fed a high fat/high sucrose diet. In a previous study, an increase in CGRP expression was observed in the TG couple hours post dural in vivo CFA administration [115]. It could therefore be interesting to test whether basal levels of CGRP in an inflammation model would rise.

There are some studies showing that corticosteroids are useful in managing resistant, severe, recurrent or prolonged migraine attacks (such as status migraenosus) in the emergency department [166]. The questions in our opinion remain as to whether these migraine cases such as status migraenosus should be considered an acute migraine or as a short chronic migraine (at least when it comes to the biology/pathology). Likewise, one could argue that if NSAIDs are effective treatment in some migraine patients, inflammation must therefore play an important pathological role. However it should be noted that these drugs have modes of actions that goes beyond their anti-inflammatory properties, such as their analgesic and antipyretic effects [167], which should be addressed in future studies.

## Data Availability

Not applicable.

## Supplementary Materials

The following supporting information can be downloaded at: https://www.mdpi.com/article/10.3390/cells11152444/s1, Table S1: Summary of pathological changes in the TG, Table S2: Summary of pathological changes in the Brain/Spinal cord, Table S3: Summary of pathological changes in the TNC.
