# Supplementary material for: Could Experimental Inflammation Provide Better Understanding of Migraines?
Source: Cells. 2022 Aug 6;11(15):2444. doi: 10.3390/cells11152444 (PMC9368653; doi:10.3390/cells11152444)
Supplement: Supplementary file 1 [file cells-11-02444-s001.zip › cells-1799438 Supplementary tables.pdf]

TG		
Pathological changes	Treatment	Treatment effective against
CGRP ↑<br>PACAP-27 ↓<br>PACAP-38 ↓<br>PGE_2 ↑<br>TRPV1 ↑<br>5-HT_7 receptor ↑<br>cAMP ↑<br>PKA ↑<br>$ERK_{1/2}$ ↑<br>CREB ↑<br>C-fos ↑<br>Neuronal sensitization<br>Mitochondrial dysfunction	Ketoprofen	CGRP, PGE_2
	Nimesulide	CGRP, PGE_2
	Etoricoxib	PGE_2
	Flunarazine	CGRP, TRPV1
	Xiongmatang Extract	CGRP, TRPV1
	Electro-acupuncture	5-HT_7 receptor, cAMP, PKA, $ERK_{1/2}$, c-fos, CREB, neuronal sensitization
	TAK-242	c-fos

Supplementary Table S1: Summary of pathological changes in the TG, Changes in the TG following IS, and the effect of experimental treatments and their potential associated targets. For detailed references we refer to the text.

BRAIN/SPINAL CORD

Pathological changes	Treatment	Treatment effective against
CGRP ↑<br>5-HT ↓<br>GABA ↓<br>GABABR1/GABABR2 ↓<br>Glutamate ↑<br>VEGF ↑<br>BDNF ↑<br>nNOS ↑<br>α7nACh receptor ↓<br>JNK ↑<br>CREB ↑<br>IL-18 ↑<br>NF-κB ↑<br>IL-1β ↑<br>TNF-α ↑<br>FKN ↑<br>CX3CR1 ↑<br>MyD88 ↑<br>TRIF ↑<br>IκB ↑<br>Microglia ↑<br>Astrocytes ↑<br>c-Fos ↑<br>Neuronal sensitization<br>Synaptic plasticity ↓<br>Changes in functional connectivity<br>Descending inhibitory pathways ↓<br>BBB permeability ↑↓<br>White matter volume ↑	Sumatriptan	VEGF
	Baclofen	CGRP, glutamate, PKA, CREB, c-fos, synaptic plasticity
	H89	CGRP, glutamate, CREB, c-fos, synaptic plasticity
	Propranolol	c-fos, neuronal sensitization
	Amitriptyline	Synaptic plasticity
	anti-IL18	IL-18, NF-κB
	PNU-282987	CGRP, α7nACh receptor, JNK, IL-18, TNF-α, microglia, astrocytes
	Electroacupuncture	Neuronal sensitization
	TAK-242	c-fos
	Wuzhuyu decoction	c-fos

Supplementary Table S2: Summary of pathological changes in the Brain/Spinal cord, Changes in the Brain/Spinal cord following IS, and the effect of experimental treatments and their potential associated targets. For detailed references we refer to the text.

<table>
<tr><th colspan="3">TNC</th></tr>
<tr><th>Pathological changes</th><th>Treatment</th><th>Treatment effective against</th></tr>
<tr><td rowspan="16">CGRP ↑
Substance P ↑
NO ↑
BDNF ↑
NGF ↑
5-HT$_7$ receptor ↓
mGluR5 ↑
P2X$_4$ receptor ↑
P2Y$_{14}$ receptor ↑
ASIC3 ↑
TNF-α ↑
IL-1β ↑
FKN ↑
CX3CR1 ↑
Microglia ↑
C-fos ↑
Neuronal sensitization
cAMP↑
PKA ↑
ERK$_{1/2}$ ↑
PKC ↑
p38 ↑
EAAT3 ↑
TrKB ↑
CREB ↑
Synaptic plasticity ↑
EphB2/EphrinB2 ↑
CaMKII ↑
Mitochondrial dysfunction
SIRT1 ↓
PGC-1α ↓
mTOR ↑
Autophagy
BBB permeability ↑↓</td><td>Sumatriptan</td><td>CGRP, 5-HT, TNF-α, IL-1β, c-fos</td></tr>
<tr><td>anti-NGF</td><td>CGRP, ASIC3, PKC, c-fos</td></tr>
<tr><td>Chelerythrin</td><td>CGRP, ASIC3, c-fos</td></tr>
<tr><td>APETx2</td><td>CGRP, c-fos</td></tr>
<tr><td>MPEP</td><td>CGRP, Substance P, mTOR, autophagy</td></tr>
<tr><td>Rapamycin</td><td>CGRP, Substance P, IL-1β, autophagy</td></tr>
<tr><td>Minocycline</td><td>IL-1β, TNF-α, microglia</td></tr>
<tr><td>Electroacupuncture</td><td>5-HT$_7$ receptor, cAMP, PKA, ERK$_{1/2}$, CREB, c-fos</td></tr>
<tr><td>EphB1-Fc</td><td>CGRP, Substance P, synaptic plasticity</td></tr>
<tr><td>TAK-242</td><td>c-fos</td></tr>
<tr><td>PP2</td><td>CGRP, Substance P, synaptic plasticity</td></tr>
<tr><td>Genistein</td><td>CGRP, Substance P, synaptic plasticity</td></tr>
<tr><td>ANA-12</td><td>CGRP, EAAT3, c-fos</td></tr>
<tr><td>TNP-ATP</td><td>CGRP, BDNF, P2X$_4$ receptor, EAAT3, p38, c-fos</td></tr>
<tr><td>SIRT1720</td><td>CGRP, Substance P, SIRT1, PGC-1α, mitochondrial dysfunction</td></tr>
<tr><td>Wuzhuyu Decoction</td><td>CGRP, TNF-α, IL-1β, c-fos</td></tr>
</table>

Supplementary Table S3: Summary of pathological changes in the TNC, Changes in the TNC following IS, and the effect of experimental treatments and their potential associated targets. For detailed references we refer to the text.
